# Baseline conditions and nutritional state upon hospitalization are the greatest risks for mortality for cardiovascular diseases and for several classes of diseases: a retrospective study

**DOI:** 10.1038/s41598-022-14643-7

**Published:** 2022-06-25

**Authors:** Lara Loreggian, Filippo Giorgini, Ahmed S. Zakaria, Marco Fanchini, Annamaria Veronelli, Antonio E. Pontiroli, Elena Tagliabue

**Affiliations:** 1 Dipartimento di Scienze della Salute, Università degli Studi di Milano, Ospedale San Paolo, Via A. di Rudinì 8, 20142 Milan, Italy; 2 International Center for T1D, Pediatric Clinical Research Center Romeo ed Enrica Invernizzi, DIBIC, Università di Milano, Milan, Italy; 3 ASST Santi Paolo e Carlo, Ospedale San Paolo, Milan, Italy; 4 IRCCS MultiMedica, Milan, Italy; 5 Present Address: Policlinico San Donato, Milan, Italy

**Keywords:** Biomarkers, Cardiology, Diseases, Health care, Medical research, Risk factors

## Abstract

The aim of this retrospective study was to evaluate risk factors for 3-years mortality after hospital discharge in all inpatients admitted to a general hospital in Milano, Italy. A total of 2580 consecutive patients admitted to Ospedale San Paolo, July 1 to December 31, 2012, for several classes of diseases (internal medicine, cancer, infectious diseases, trauma and surgery, pneumonia, and heart diseases) were studied. Age, total disease, type of admission, length of admission, age-adjusted Charlson index, prognostic nutritional index (PNI), and full blood count were evaluated. Univariate Cox models were used to evaluate the association between variables and death. Of the 2580 consecutive patients (age 66.8 ± 19.36 years, mean ± SD), 920 died within 3 years after discharge. At univariate analysis, all investigated variables, except sex and lymphocytes, were associated with patient death. Stepwise regression analyses revealed that the age-adjusted Charlson index or age plus total diseases, type of admission, number of admissions, and PNI were significant risk factors in the whole sample and in some classes of disease. Results were superimposable when considering death from date of admission instead of date of discharge, meaning that in-hospital death was not relevant to the total death count (115 out of 902). Seriousness of baseline conditions represents the major risk factor for mortality in most classes of disease, and possibly influences other predictors, such as type of admission and length of stay. This suggests that the current model of hospital admission might be improved, for instance, through comprehensive care at home, instead of hospital admission, or before admission.

## Introduction

In recent years, a series of studies have evaluated survival after hospital admission as a possible risk factor for long-term mortality^1–18^. In addition to age, which is a universal strong and unmodifiable risk factor for mortality in men and women^1–18^, additional risk factors have been identified for selected diseases, such as heart failure (CHF)^5,19,20^, sepsis and acute and chronic pulmonary diseases^3,6,9,14^, surgery^8,10^, hip fractures^17^, and stroke^6,7,12^. For instance, in CHF and hip fracture, male sex is important^5,17^; in the elderly, pre-admission conditions are a major risk factor for long-term mortality, as well as admission to an intensive care unit (ICU), type of admission (emergency vs. elective), presence of comorbidities, diabetes, and chronic kidney disease^1,2,4,9,11,13,15,18,20^. In patients with acute and chronic pulmonary diseases, male sex, comorbidities, long in-stay, creatinine, and albumin are risk factors^3,6,9,14^. Even though it might be logical to assume that the same risk factors apply to all the above conditions as well as to other diseases, this has not been demonstrated. For patients admitted for neurological diseases, better long-term survival has also been associated with full assistance after discharge, and social factors have been shown to predict mortality, mainly mediated by poor assistance after discharge^11,15^.

Upon recognition of poor nutritional status as one of the major reasons for prolonged in-hospital stay^21^, malnutrition is considered a risk factor for long-term mortality, and a poor nutritional index is a marker of poor prognosis in patients with cardiovascular diseases^1,3,13^, and in patients with cancer^22^. A few years ago, in a general hospital in the Lombardy region, we found that low baseline levels of albumin and lymphocytes were associated with prolonged in-hospital stay for 4000 patients, regardless of the disease or the condition for admission^21^. In the present study, we evaluated survival of the same patients after discharge, and the prognostic risk factors for 3-years mortality, including clinical and laboratory variables and nutritional state, in the whole population and in selected classes of diseases.

## Patients and methods

Details of the original study have been previously published^21^. During the second half of 2012 (1st July to December 31, 2012), we considered all subjects admitted as inpatients at Ospedale San Paolo, Milan, Italy, in the following wards: surgery (2 wards), obstetrics and gynecology, infectious diseases, medicine (three wards), orthopedics, and urology (total number of beds at that period = 229). Patients admitted as outpatients at Day Hospital were excluded from the original study because the aim was to test how low levels of albumin and lymphocytes influenced the length of stay. Data collection was performed through electronic charts, recording anamnestic data such as gender, age, date of admission and discharge, type of diagnosis, concomitant diseases (identified by the International Classification Code of Diseases), and biochemical indices such as complete blood count with lymphocyte count and albumin measured during the first 24 h. For this follow-up study, the same cohort of patient was analyzed and classes of diseases were grouped as follows, based on the primary diagnosis: internal medicine (non-communicable diseases of the gut, liver, kidneys, hematological disorders, cerebrovascular diseases, lungs with the exclusion of pneumonia and heart diseases), malignant neoplasia (solid tumors, lymphoproliferative, leukemia), infectious diseases (AIDS, TBC, systemic infections, sepsis), all traumatic and surgical patients, pneumonia, heart diseases (infarction, heart failure), and other diseases (acute poisoning, anaphylaxis, psychosis). The ICD9 codes used are listed in Supplemental Table 1.

The amount of concomitant diseases (diagnoses) was also considered as “total number of diseases”. The type of admission was included as elective vs. emergency admission through the emergency department. We identified presence of diabetes either if it was specifically stated in the discharge cards or in presence of blood glucose > 160 mg/dl. We decided to use a different value from the standard threshold of 123 mg/dl, since it was not possible to state if blood examinations had always been performed under fasting conditions. For this reason, analyses were also performed using an alternative threshold of blood glucose greater than 200 mg/dl. We also calculated the age-adjusted Charlson Index, which yields a severity score of a patient based on different ages and presence of concomitant diseases^23,24^.

### Laboratory analysis

All variables were evaluated using standardized mass methods on automatic machinery; blood glucose, liver enzymes (AST, ALT, µGT, ALP, bilirubin, and cholinesterase), full blood count, creatinine, calcium, phosphorus, sodium, potassium, uric acid, C-reactive protein (CRP), proteins, and albumin) were assessed. The prognostic nutritional index (PNI) was calculated as follows: PNI = 10 × serum albumin (g/dL) + 0.005 × total lymphocyte count (per mm^3^)^19^. The frequency of examinations performed during the first 24 h is reported in Supplemental Table 2, divided by ward, by class of disease, and by type of admission. Supplemental Table 2a also reports all examinations performed per patient during inpatient stay.

### Procedures

As shown in the flowchart (Supplemental Fig. 1), patients were identified through personal identification codes entered into the Regional Lombardy Administrative Database; thus, it was possible to ascertain whether patients were alive, or dead, or had moved to other regions. The National Health System (NHS) covers more than 95% of all hospital admissions, medical and surgical procedures, and medical expenses of citizens^25^ (Italian Survey 2012). The Regional Lombardy Administrative Database contains all pertinent data of all citizens since 1988, independent of participation in studies and loss to follow-up. In particular, the Lombardy database collects several pieces of information, including (1) an archive of residents who receive NHS assistance, reporting demographic and administrative data; (2) a database on diagnosis at discharge from public or private hospitals in the whole region; (3) a database on outpatient drug prescriptions reimbursable by the NHS; and (4) a database on outpatient visits, including specialist ambulatory care and diagnostic laboratories accredited by the NHS. For each patient, these databases were linked using a single identification code. This procedure has already been employed and validated in previous studies in Lombardy, Italy^26,27^. The date of death was collected for each patient, and live subjects were censored on the conventional date of March 15, 2016. Subjects without information about outcome (dead/alive at that date), migrated subjects and subjects who could not be retrieved were excluded. Since women admitted for pregnancy and delivery had only one death out of 1006, women with this diagnosis were excluded from the study.

### Statistical analysis

Data are shown as average values (± SD) for continuous variables or as absolute numbers and percentages for discrete variables. Continuous variables were compared using the Student’s t-test or the non-parametric Wilcoxon test. Frequencies were compared using the chi-square test or the Fisher exact test. Univariate Cox models were used to evaluate the association between variables and death in the whole sample and stratified for different diseases. Hazard Ratios (HR) with 95% confidence intervals (CI) and p-values were calculated. Two stepwise regression models of risk factors for mortality were performed (Cox proportional hazards models) to identify the best multivariable models. All significant variables at the univariate analysis, observed in almost 50% of subjects, were entered into the two models. To avoid collinearity between variables, the first model (model 1) included the age-adjusted Charlson index, while the second model (model 2) included age and total diseases. Statistical analyses were performed using SAS Software 9.4, and STATA 12.0, for MacIntosh. For all statistical analyses p-values < 0.05 were considered statistically significant. This manuscript was prepared following the guidelines of the STROBE statement^28^ (Supplemental File 1).

### Ethical approval

Comitato Etico Ospedale San Paolo, Milan, Italy n. 918, 24.10.2013, original study; Comitato Etico Area 1 Milan, Italy n. 222, 23.03.2021. The research was performed in accordance with the relevant guidelines and regulations. Being a retrospective cohort study, informed consent was obtained from all individual participants included in the study, who could be reached by interview, phone, or letter.

## Results

After exclusion of women admitted for pregnancy and delivery (1 death out of 1006 women), 2580 patients were considered, with follow-up of 934.3 ± 489.08 [915.40–953.17] days (mean ± SD [95% CI]), range 1–1334 days. Of the 920 dead patients 483 were men and 437 were women (not significant). Table 1 shows baseline details of the patients, including age, sex, number of deaths for each primary diagnosis, age-adjusted Charlson index, PNI and laboratory analyses. Patients with more than one admission during the study period were 135/2580 (5.2%, not shown). Diseases with greater percentage of deaths were cardiovascular and pneumonia, followed by neoplasia. Table 2 shows the frequency of comorbidities. For each class of disease around 90% of patients had up to three co-morbidities (i.e., three diagnoses) with no significant differences among classes. In contrast, the age-adjusted Charlson Index yielded significant differences among classes of disease (p < 0.0001, not shown) with higher values for neoplasia and cardiovascular, and lower values for trauma-surgery. Patients differed according to the type of admission, elective vs. emergency (Supplemental Table 3), with the latter being more acutely and chronically unhealthy. Some variables were expressed both as continuous and in a dichotomous way (above and below the mean value of the cohort, age-adjusted Charlson Index as quartiles); their significance at univariate analysis did not change.

**Table 1:** Baseline characteristics of patients by classes of diseases.

Variable	Classes of disease (primary diagnosis)							
	All patients	Internal medicine	Neoplasia	Infectious diseases	Trauma surgery	Others	Pneumonia	Cardio-vascular
N patients	2580	556	522	327	717	120	150	188
**Sex**								
M	1317 (51%)	271 (48.74%)	286 (54.79%)	178 (54.43%)	357 (49.79%)	58 (48.33%)	84 (56%)	83 (44.15%)
F	1263 (49%)	285 (51.26%)	236 (45.21%)	149 (45.57%)	360 (50.21%)	62 (51.67%)	66 (44%)	105 (55.85%)
Age	66.8 ± 19.36	69.6 ± 17.28	68.7 ± 14.08	60.2 ± 21.03	59.8 ± 21.19	68.8 ± 22.59	76.7 ± 15.35	82.7 ± 9.71
**Status**								
Alive at 3 years	1660 (64.34%)	347 (62.41%)	292 (55.94%)	209 (63.91%)	617 (86.05%)	74 (61.67%)	56 (37.33%)	65 (34.57%)
M/F	834/826	170/177	139/153	121/88	306/311	38/36	33/23	27/38
Dead at 3 years	920 (35.66%)	209 (37.59%)	230 (44.06%)	118 (36.09%)	100 (13.95%)	46 (38.33%)	94 (62.67%)	123 (65.43%)
M/F	483/437	101/108	147/83	57/61	51/49	20/26	51/43	56/67
Charlson index	3.9 ± 2.41	3.7 ± 1.69	5.7 ± 2.97	3.7 ± 2.63	2.5 ± 1.72	3.4 ± 2.01	4.1 ± 1.55	5.1 ± 0.84
Duration of in-stay	8.8 ± 6.55	8.2 ± 5.09	8.9 ± 6.60	9.9 ± 6.42	8.7 ± 8.00	6.5 ± 6.03	10.7 ± 6.21	8.4 ± 3.47
PNI	32.4 ± 6.50	32.5 ± 6.09	31.7 ± 6.84	31.0 ± 6.23	35.9 ± 7.35	34.8 ± 7.17	29.8 ± 5.11	32.3 ± 4.45
Creatinine	1.1 ± 1.06	1.3 ± 1.24	1.0 ± 0.73	1.1 ± 1.10	1.0 ± 1.18	1.1 ± 0.95	1.2 ± 0.79	1.4 ± 0.92
ALP	113.5 ± 108.59	116.6 ± 116.03	140.5 ± 165.27	110.3 ± 80.44	123.4 ± 156.97	90.9 ± 98.11	104.8 ± 62.39	95.2 ± 38.74
gGT	81.6 ± 133.91	79.9 ± 139.04	106.3 ± 196.04	96.2 ± 145.47	87.4 ± 113.78	50.5 ± 54.62	65.5 ± 75.83	58.3 ± 65.62
CHE	5219.1 ± 2112.64	5592.2 ± 2039.45	4727.5 ± 2045.43	4681.2 ± 2341.74	5553.9 ± 2102.08	6051 ± 1749.53	4687.6 ± 1559.57	5871.9 ± 1987.33
Calcium	8.3 ± 0.65	8.4 ± 0.68	8.3 ± 0.68	8.3 ± 0.65	8.4 ± 0.57	8.5 ± 0.65	8.3 ± 0.85	8.3 ± 0.50
HGB	12.3 ± 2.23	12.5 ± 2.32	11.9 ± 2.22	12.2 ± 2.17	12.8 ± 2.09	12.1 ± 2.30	12.2 ± 2.03	11.7 ± 2.31
RBC	4.2 ± 0.88	4.3 ± 0.79	4.1 ± 1.16	4.1 ± 0.76	4.4 ± 0.82	4.2 ± 0.77	4.1 ± 0.71	4.1 ± 0.82
HCT	36.8 ± 6.10	37.3 ± 6.42	35.6 ± 6.05	36.1 ± 5.99	37.9 ± 5.55	36 ± 6.06	36.9 ± 5.90	36.0 ± 6.64
MCV	88.0 ± 8.44	88.4 ± 8.51	87.4 ± 8.50	88.3 ± 8.74	87.2 ± 7.96	86.4 ± 9.03	90.1 ± 7.82	89.5 ± 8.73
WBC	10.5 ± 6.06	10.2 ± 4.58	10.7 ± 7.63	11.0 ± 7.01	10.3 ± 4.08	8.9 ± 3.70	12.7 ± 8.42	9.8 ± 7.41
Lymphocytes	1.6 ± 2.46	1.7 ± 1.31	1.6 ± 2.94	1.6 ± 1.72	1.6 ± 0.77	1.7 ± 0.85	1.4 ± 1.19	2.0 ± 6.54
Albumin	3.2 ± 0.65	3.2 ± 0.61	3.2 ± 0.68	3.1 ± 0.62	3.6 ± 0.73	3.5 ± 0.72	3.0 ± 0.51	3.2 ± 0.45
Blood glucose	106.7 ± 43.78	111.7 ± 59.20	111.5 ± 40.87	101.3 ± 42.25	107.0 ± 33.22	92.4 ± 28.46	104.8 ± 40.38	100.2 ± 34.39

Numbers are absolute frequencies (%) or means ± standard deviation. Charlson index is age-adjusted.

*M* male, *F* female, *PNI* prognostic nutritional index, *ALP* alkaline phosphatase, *gGT* gamma-glutamil-transpeptidase, *CHE* cholinesterase, *HGB* hemoglobin, *RBC* red blood cells, *HCT* hematocrit, *MCV* mean corpuscular volume, *WBC* white blood count.

**Table 2:** Number of diseases and age-adjusted Charlson index for each class of diseases.

Class of diseases (primary diagnosis)	Number of diseases (diagnoses)							Total	Charlson index*
	1	2	3	%	4	5	6		
Internal medicine	205	165	146	92.8	34	4	2	556	3.7 ± 1.69
Neoplasia	167	164	141	90.4	36	10	4	522	5.7 ± 2.97
Infectious	107	94	98	91.4	22	4	2	327	3.7 ± 2.63
Trauma-surgery	374	197	108	94.7	25	10	3	717	2.5 ± 1.72
Others	52	41	23	96.7	4	0	0	120	3.4 ± 2.01
Pneumonia	20	53	67	93.3	9	0	1	150	4.1 ± 1.55
Cardiovascular	56	60	66	96.8	6	0	0	188	5.1 ± 0.84
Total patients	981	774	649	93.3	136	28	12	2580	3.9 ± 2.41

*Mean ± standard deviation; % indicates percentage of patients with up to three diagnoses.

Charlson index is age-adjusted.

Table 3 shows risk factors for the whole sample and for each primary diagnosis. At Cox univariate analysis, both clinical conditions (age, total diseases, kind of admission, duration of in-stay, age-adjusted Charlson Index) and metabolic parameters (creatinine, albumin, full blood count, PNI) were associated with mortality in the whole population, except sex and lymphocytes. Furthermore, some variables were significant in selected classes of diseases, but not in the whole sample, and sometimes the opposite occurred. Details of Hazard Ratios with 95% CI and p-values were reported in Supplemental Table 4. The presence of diabetes, as well as blood glucose levels, were of reduced interest, however, patients with diabetes were more frequent among emergency then among elective admissions (79.7% vs 20.3%, p = 0.001, Supplementary Table 3). Supplemental Table 5 shows that age-adjusted Charlson index was always significantly higher in dead patients than in alive patients in all classes of disease.

**Table 3:** Univariate analysis of risk factors for 3-years mortality in the whole sample and in different classes of disease.

	All patients	Classes of disease (primary diagnosis)						
		Internal medicine	Neoplasia	Infectious diseases	Trauma-surgery	Others	Pneumonia	Cardiovascular
Number of patients	2580	556	522	327	717	120	150	188
**Risk factors**								
Type of admission (urgency)	3.26***	3.21***	4.81***	2.66**	3.56***	1.65	0.77	0.94
Total diseases	1.46***	1.36***	1.45***	1.31**	1.60***	1.35	1.22	1.28*
Age-adjusted Charlson index	1.34***	1.56***	1.35***	1.16***	1.84***	1.91***	1.28***	1.35**
Number of admissions	1.45***	0.98	1.20	1.76*	2.36*	0.82	2.45**	1.00
Sex (female)	0.94	1.04	0.62***	1.35	0.95	1.28	1.20	0.93
Age	1.06***	1.06***	1.05***	1.05***	1.08***	1.08***	1.04***	1.04***
Duration of in-stay (days)	1.03***	1.02	1.04***	1.01	1.05***	0.98	1.00	1.03
Creatinine^^^	1.01***	1.01***	1.02*	1.01*	1.01	1.00	1.02	1.02*
ALP^^^^	1.02***	1.01	1.02***	1.03*	1.02	1.06**	1.03	1.03
gGT^^^^	1.01***	1.01	1.01**	1.02***	1.01	1.01	1.01	0.96
CHE^^^^	0.99***	0.99	0.99*	0.99***	0.99**	0.99	0.99	0.99
Calcium	0.64***	0.57***	0.71**	0.62**	0.71	0.42*	1.17	0.42***
HGB	0.86***	0.87***	0.93**	0.81***	0.90*	0.93	0.89*	0.87***
RBC	0.65***	0.60***	0.92	0.56***	0.59***	1.06	0.73*	0.71**
HCT	0.96***	0.96***	0.97*	0.94***	0.97	1.00	0.97	0.95***
MCV	1.03***	1.04***	1.01	1.03**	1.08***	0.99	1.03	1.00
WBC	1.03***	0.99	1.03***	1.02*	1.02	1.11*	1.04***	1.00
Lymphocytes	1.01	0.88	1.02	0.89	0.64**	0.65	1.08	1.03*
Albumin	0.52***	0.44***	0.76*	0.39***	0.51***	0.40**	0.53**	0.49**
Blood Glucose	1.02*	1.01	1.00	1.04	1.04	1.11*	1.04	1.02
PNI	0.94***	0.92***	0.97*	0.91***	0.94***	0.91**	0.94**	0.93**
Diabetes (BG > 160 mg/dl)^§^	1.53***	1.20	1.43	1.59	2.14*	1.88	1.64	1.24
Diabetes (BG > 200 mg/dl)^§§^	1.49**	1.10	3.14***	1.32	1.86	N.E	1.20	0.83

Data are presented as Hazard Ratios (HR); significance.

HR for continuous variables were calculated for 1-unit increase; ^^^HR for 10-unit increase; ^^^^HR for 0.1-unit increase; ^§^Diagnosis of diabetes based on blood glucose levels > 160 mg/dl (plus subjects identified in the charts); ^§§^Diagnosis of diabetes based on blood glucose levels > 200 mg/dl (plus subjects identified in the charts); *p < 0.05; **p < 0.01; ***p < 0.001.

*ALP* alkaline phosphatase, *gGT* gamma-glutamil-transpeptidase, *CHE* cholinesterase, *HGB* hemoglobin, *RBC* red blood cells, *HCT* hematocrit, *MCV* mean corpuscular volume, *WBC* white blood count, *PNI* prognostic nutritional index, *BG* blood glucose, *N.E.* not estimable.

**Figure 1:**
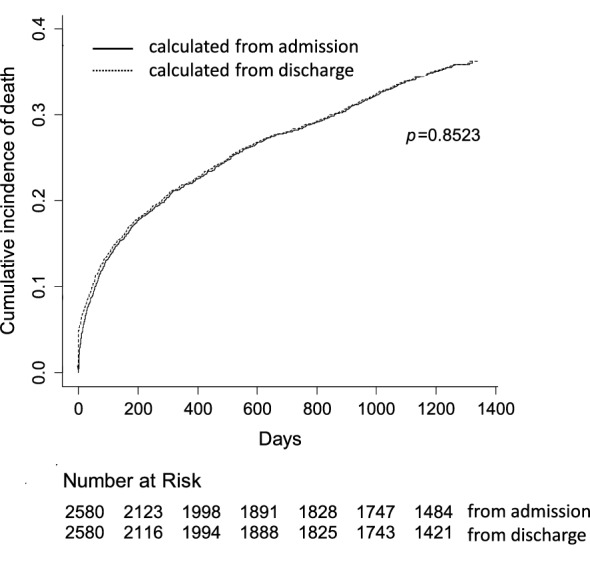
Cumulative incidence of mortality calculated from date of admission and from date of discharge. Patients at risk for each curve are shown.

Various multivariable models were implemented using clinical conditions and metabolic variables. Since total diseases plus age and age-adjusted Charlson index represented the same components, total diseases and age were used alternatively to age-adjusted Charlson index. In the stepwise regression analyses, either age-adjusted Charlson index (model 1) or age plus total diseases (model 2) were entered into the model, together with significant variables at univariate analysis (Table 4). We found that for the whole population, the best model included age-adjusted Charlson index, number of admissions, type of admission, PNI, and hemoglobin. When age plus total diseases were used in place of age-adjusted Charlson index, together with number of admissions, type of admission, PNI, and hemoglobin, we found that total diseases was no more significant for the whole population. Multivariable Cox regression models were performed for each classes of disease by using variables resulted by stepwise regression for the whole population. Considering model 1, it appears that the age-adjusted Charlson index was significant for all classes of disease. In contrast, the type of admission and number of admissions were significant in four and three classes of diseases, respectively; PNI and hemoglobin were significant in four and two classes of diseases, respectively. Similar results were obtained in the model 2 (age and total diseases instead of age-adjusted Charlson index): age was significant for all classes of diseases, except for neoplasia, while the other variables were significant in some classes. Details of HR (95% CI) and p-values were reported in Supplemental Table 6. Finally, the results were superimposable when the time to death was calculated from the date of admission rather than from the date of discharge (Fig. 1); the three years (1095 days) cumulative incidence of dead patients was 33.80% and 33.99%, respectively (p = 0.853, NS).

**Table 4:** Multivariable Cox regression models to evaluate variables predicting mortality.

	All patients	Classes of disease (primary diagnosis)						
		Internal medicine	Neoplasia	Infectious diseases	Trauma-surgery	Others	Pneumonia	Cardiovascular
**Model 1**								
Age-adjusted Charlson index	1.25***	1.34***	1.21***	1.14**	1.72***	1.77***	1.22*	1.37*
Type of admission (urgency)	2.41***	1.59	2.90***	4.83**	3.87*	0.86	0.76	0.55
Number of admissions	1.41**	1.39	1.00	1.65	2.28*	1.35	2.59*	1.14
PNI	0.95***	0.93***	0.98	0.94**	0.96	0.96	0.96	0.95*
Hemoglobin	0.94**	0.98	0.99	0.83***	0.99	1.04	0.91	0.92
**Model 2**								
Age	1.04***	1.05***	1.01	1.04***	1.07***	1.08***	1.02*	1.03*
Type of admission (urgency)	1.41**	1.42	2.88***	2.05	1.63	0.65	0.83	0.65
Number of admissions	1.49**	1.32	0.94	2.24**	4.58***	1.39	2.57*	1.11
PNI	0.96***	0.93***	0.98	0.95*	0.97	0.96	0.96	0.96
Hemoglobin	0.94**	0.98	0.97	0.87**	0.98	1.04	0.90	0.91

Data are presented as Hazard Ratios (HR); significance.

HR for continuous variables were calculated for 1-unit increase.

Showed variables were selected as best model from two stepwise regression models in the whole population. To avoid collinearity, model 1 included age-adjusted Charlson index, while in model 2 age plus total diseases were used (total diseases was not selected from the stepwise procedure). Selected variables in the whole population were tested as multivariable models for each class of disease.

*PNI* prognostic nutritional index. *p < 0.05; **p < 0.01; ***p < 0.001.

## Discussion

Various clinical and metabolic risk factors have been reported for long-term mortality after hospital admission, depending on patient age and type of admission. In addition, baseline conditions prior to admission were risk factors for mortality. Of interest, of the many studies published, the majority is focused on selected classes of diseases^1–20^, leading to the assumption that the risk factors identified only apply to selected diseases. In our study, most patients showed two or more comorbidities at admission, indicating that most patients were not admitted simply for an acute disease. In our study, most risk factors applied to most diseases to a similar degree. For instance, age and age threshold, calcium, albumin, PNI, lymphocyte threshold, and age-adjusted Charlson Index were almost universal risk factors. In multivariable analysis, various models of mixed clinical and laboratory variables predicted mortality, although to different degrees in the different classes of diseases.

Notably, diabetes and blood glucose levels were of little significance; this is in contrast with a recent study suggesting that blood glucose levels at admission are linked to 10-y mortality in non-diabetic patients^29^. The reasons for this difference could be the duration of follow-up and types of patients under study, as in our study, diabetes was mainly a secondary diagnosis. In contrast, diabetes was more frequent among patients with emergency admission and was associated with longer in-hospital stay, similar to what has been found in a recent study in Spain^30^.

Various risk indexes have been proposed to identify subjects at high risk of hospital complications, and it has also been shown that in-hospital mortality is correlated with long-term mortality^1,23,24^. We do not provide a new prognostic index, even though we employed the consolidated Charlson Index^23,24^ and PNI^19^. We suggest that the main risk factors for mortality after in-hospital admission are seriousness of baseline conditions, including malnutrition. and that the majority of risk factors for mortality apply to several classes of diseases, not only to selected diseases. For instance, the fact that total diseases or type of admission are risk factors for mortality does not depend on hospitalization, but on the patient’s story, as it is for malnutrition (low PNI) that also correlates with length of stay^21^. From the results obtained, the seriousness of baseline conditions seems to be the main determinant of mortality, with little influence of in-hospital stay. For example, pregnant women, younger than the other classes of diseases/conditions, had a very low mortality rate (1 out of 1006), and were excluded from the study. In addition, superimposable results were obtained when death was calculated from the date of admission instead of from the date of discharge, indicating that mortality during in-hospital stay does not contribute significantly to total death count.

This study had several limitations. Being monocentric, the study reflects the admissions and discharges of a single general hospital in Lombardy. In addition, since analyzed subjects came from an original study aimed to investigate the role of malnutrition at admission in the length of stay, only examinations performed during the first 24 h were collected. This does not mean that other examinations were not performed, as the average number of blood examinations per in-patient was 63.1 (Supplementary Table 2a), which was not different from the average of exams performed in other general hospitals in Lombardy (Epidemiology Observatory—Department of Health of Lombardy Region, Milan, Italy). Depending on the habits of the different departments, other examinations (blood lipids, uric acid, alkaline phosphatase, cholinesterase, bilirubin, phosphorus, iron, ferritin, vitamin B12, folate, hba1c) were performed in less than half of the patients within the first 24 h; these examinations were not considered and were not significantly related to either length of stay or status. Another limitation is that we could only evaluate the date of death, with no details on the cause of death.

It is worth to consider that unplanned admission may be functionally and psychologically deleterious for older people^16^, that hospital admission may be followed by life-long follow-up visits, and the lack of this post-hospital coverage may be an additional poor prognostic factor^11,15^. This leads to the question of adequacy of the current hospitalization procedure; taking into account all the possible benefits and negative consequences of in-hospital stay, we should consider alternatives for patients, such as domiciliary comprehensive care. This does not mean to let compromised patients unattended at home, but just the opposite: efforts should be made to improve patients’ health conditions and wellbeing in general, and the nutritional state in particular. To date this alternative can be pursued, and it is probably less costly and more effective than traditional in-hospital admission. Future research is required to substantiate this hypothesis by comparing the outcomes of patients admitted to hospitals and patients comprehensively treated at home^31^.

Finally, this study was conducted during the pre-Covid era. However, recent studies indicate that survival after Covid is linked to age, comorbidities, Charlson Index, and nutritional status (low albumin levels and low lymphocyte count)^32–35^.

## Conclusions

Our results show that age, albumin, PNI, and age-adjusted Charlson Index were universal risk factors for mortality in all patients and in several classes of diseases. This implies that the seriousness of baseline conditions is the main determinant of mortality, with little influence on in-hospital stay. This leads to the question of adequacy of the current procedure to admit all patients as in-patients; one should consider alternatives, such as comprehensive care, at the best possible levels, at home; efforts should be made to improve health conditions in general, and the nutritional state in particular. This alternative is now possible to pursue, and it is probably less costly and more effective than traditional in-hospital admission.

## Supplementary Information

Supplementary Information.

## Data Availability

All datasets generated and analyzed during the current study are not publicly available but are available from the corresponding author upon reasonable request.

## Abbreviations

CHF: Heart failure
ICU: Intensive care unit
AIDS: Acquired immune deficiency syndrome
TBC: Tuberculosis
AST: Aspartate aminotransferase
ALT: Alanine aminotransferase
µGT: Gamma-glutamil-transpeptidase
ALP: Alkaline phosphatase
PNI: Prognostic nutritional index
NHS: National health system
