# Supplementary material for: Baseline conditions and nutritional state upon hospitalization are the greatest risks for mortality for cardiovascular diseases and for several classes of diseases: a retrospective study
Source: Sci Rep. 2022 Jun 25;12:10819. doi: 10.1038/s41598-022-14643-7 (PMC9233677; doi:10.1038/s41598-022-14643-7)
Supplement: Supplementary file 1 — Supplementary Information. [file 41598_2022_14643_MOESM1_ESM.docx]

Supplementary Appendix

Baseline conditions and nutritional state upon hospitalization are the greatest risks for mortality for cardiovascular diseases and for several classes of diseases; a retrospective study

Lara Loreggian, Filippo Giorgini, Ahmed S Zakaria, Marco Fanchini, Annamaria Veronelli, Antonio E. Pontiroli, Elena Tagliabue

Flowchart of the study.

Analysis of baseline conditions,

nutritional state, kind of admission, number of admissions in alive vs dead patients

Hospital admission

in Milan, Italy, period 07.01.2012-12.31.2012

Admission Records

(N = 3586)

Excluded

Pregnancy and delivery

N = 1006 (1 death)

Alive or dead at 15.03.2016

(Lombardy Regional Database)

921 dead patients

Considered for study

All patients affected by

non-communicable diseases, cancer, traumatic and surgical diseases, pneumonia, heart diseases, and miscellaneous diseases

N = 2580 (920 deaths)

**Supplemental Fig. 1**

**Supplemental Table 1.** ICD9 codes used for the classes of diseases *

**Internal Medicine**

430 431 586 591 986 1398 2452 2720 2749 2777 2800 2811 2830 29093314 3330 3452 3682 4011 4019 4321 4329 4359 4370 4377 4538 4556 4928 5401 5409 5562 5565 5566 5570 5589 5602 5641 5693 5711 5712 5718 5719 5722 5732 5733 5750 5761 5764 5771 5813 5852 5853 5854 5855 5856 5920 5921 5941 5949 5997 6010 6011 6040 6088 6140 6141 6145 6149 6159 6173 6191 6201 6202 6266 6826 7868 7880 9642 9720 23875 24290 25002 25032 25060 27801 28262 30002 33523 34510 34691 34982 37730 37732 43491 45111 45620 49301 49302 49321 51883 51884 53012 53100 53110 53200 53500 53501 53540 53541 56081 56212 56721 57400 57401 57410 57420 57431 57450 57451 57461 57471 58281 60001 60490 70710 71430 73007 75311 78321 78609 78820

**Neoplasia**

035 042 154 179 185 193 220 388 389 414 1501 1508 1510 1512 1514 1518 1519 1520 1530 1531 1532 1533 1534 1535 1536 1537 1539 1540 1541 1543 1550 1560 1561 1570 1571 1572 1578 1579 1588 1623 1629 1716 1729 1735 1744 1749 1809 1820 1830 1882 1889 1890 1891 1892 1893 1911 1915 1919 1958 1960 1961 1972 1976 1977 1983 1985 1990 1991 2111 2113 2114 2115 2116 2136 2141 2180 2181 2182 2189 2230 2270 2304 2350 2376 2384 2630 2769 2851 4189 4572 4589 4590 4660 4784 4821 5119 5185 5715 5770 5772 5845 5951 5954 5981 5982 5997 6183 6210 6266 7806 7863 7876 7885 8080 9599 20202 20280 20287 20300 20410 20500 20510 20800 22809 23691 23873 27651 28522 28983 41519 43401 46619 47834 48239 49121 51881 53560 55221 55321 56039 56081 56211 56212 56721 56941 56981 57420 57451 57490 59081 60090 78559 80703 82013 82020 99831 V1005 V1006 V1011 V103 V1051 V1082 V1083 V442 V443 V5811

**Infectious Diseases**

0021 075 0085 0091 0092 0310 0319 0380 0382 0389 0413 414 416 462 463 566 570 785 830 841 845 1014 1103 1113 1123 1214 1725 1726 2450 2798 2893 3208 3209 3310 3312 3810 3811 3819 3842 3849 4100 4109 4111 4119 4210 4660 5109 5119 5227 5273 5311 5550 5559 5582 5589 5609 5693 5715 5720 5781 5853 5902 5949 5990 6145 6868 6869 7020 7030 7031 7051 7080 7280 7806 9881 11284 20218 27651 28800 30000 34510 34540 41519 43882 45119 46619 48284 51884 53551 53783 56039 56723 56981 57140 57491 59010 59080 60490 64663 64683 64761 64893 65801 66624 68100 70703 70711 71106 71198 73000 73002 73008 73010 73012 73098 99591 99665 99667 V290 V711 V712

**Trauma-Surgery**

220 470 591 1550 1890 2554 2761 2800 2851 3542 3840 4472 4552 4556 4564 4589 4590 4610 5300 5307 5373 5400 5401 5409 5430 5521 5531 5538 5550 5568 5570 5602 5650 5651 5679 5691 5712 5715 5738 5762 5770 5771 5772 5854 5855 5902 5920 5921 5942 6012 6141 6169 6170 6171 6178 6183 6185 6200 6201 6202 7172 7242 7243 7350 8024 8052 8054 8133 8170 8221 8240 8242 8244 8246 8250 8260 8472 8793 8794 8832 8912 9052 9053 9054 9472 9982 27801 33520 36181 36511 47412 53020 53085 53130 53200 55000 55010 55090 55091 55092 55093 55121 55201 55221 55229 55301 55321 56039 56210 56211 56212 56402 56402 56941 56983 56985 57400 57401 57410 57420 57451 57471 57511 59001 59389 60000 60001 60011 60820 61801 61804 62130 64761 64763 66971 70703 71515 71516 71525 71783 72402 72664 72760 72763 72767 73016 73301 73315 73342 73382 80702 80842 81000 81100 81200 81201 81212 81221 81231 81240 81301 81305 81321 81340 81341 81342 81500 81610 82001 82002 82003 82010 82011 82013 82020 82021 82022 82100 82101 82300 82302 82310 82320 82321 82322 82340 82382 82521 82525 83101 83802 83809 85200 85400 86412 86503 86510 92401 99642 99666 99667 99811 99812 99831 V4364 V553

**Various Others**

261 340 605 725 937 2512 2760 2761 2767 2768 2801 2809 2818 2841 2851 2859 2875 2903 2922 2981 3320 3341 3564 3950 5609 5789 6255 7820 9351 9663 9694 9950 27652 28731 30501 34600 36101 38610 43821 53081 53240 64830 72889 78001 78009 78650 78900 79093 94219 96509 V4569

**Pneumonia**

481 485 486 2761 4821 4829 4830 5070 5849 7963 48283 51881

**Cardiovascular Diseases**

2800 4259 4280 4281 5849 7802 28529 41002 41011 41090 41091 41189 41519 42731 42732 42760 43401 51881 53200 56210

* for subjects with a surgical intervention for neoplasia the ICD9 used was neoplasia

**Supplemental Table 2.** Frequency of blood examinations performed during the first 24 h, by ward (2a), by class of disease (2b), and by type of admission (2c)

Supplemental Table 2a. Frequency of blood examinations performed during the first 24 h, by ward. The column on the far right reports the mean of all examinations performed per patient during the in-patient stay and the total average.

| Ward | patients | BG | Crea | AST | ALT | Ca | Na | K | CRP | Hgb | Albumin | Total | Per patient | All |
| --- | --- | --- | --- | --- | --- | --- | --- | --- | --- | --- | --- | --- | --- | --- |
| Surgery | 657 | 426 | 451 | 266 | 263 | 261 | 428 | 444 | 185 | 621 | 222 | 3567 | 5.43 | 48.8 |
| Ob Gyn | 132 | 35 | 35 | 34 | 31 | 13 | 34 | 31 | 26 | 112 | 4 | 355 | 2.69 | 14.9 |
| Infectious dis | 181 | 175 | 173 | 172 | 172 | 157 | 166 | 166 | 124 | 181 | 129 | 1615 | 9.02 | 142.2 |
| Medicine | 1105 | 854 | 955 | 682 | 684 | 569 | 910 | 903 | 576 | 1096 | 822 | 8051 | 7.28 | 88.6 |
| Orthopedics | 264 | 117 | 170 | 59 | 60 | 128 | 166 | 166 | 49 | 219 | 67 | 1201 | 4.54 | 36.5 |
| Urology | 241 | 119 | 178 | 55 | 56 | 124 | 175 | 175 | 67 | 189 | 32 | 1160 | 4.81 | 47.7 |
| Total | 2580 | 1726 | 1962 | 1268 | 1266 | 1252 | 1879 | 1885 | 1027 | 2418 | 1276 | 15959 | 6.18 | 63.1 |

Supplemental Table 2b. Frequency of blood examinations performed during the first 24 h, by class of disease

| Class of disease | patients | BG | Crea | AST | ALT | Ca | Na | K | CRP | Hgb | Albumin | Total | Per patient |
| --- | --- | --- | --- | --- | --- | --- | --- | --- | --- | --- | --- | --- | --- |
| Internal med | 556 | 384 | 448 | 291 | 292 | 272 | 419 | 424 | 260 | 536 | 331 | 3597 | 6.47 |
| Cancer | 522 | 357 | 383 | 232 | 232 | 269 | 384 | 382 | 147 | 473 | 256 | 3115 | 5.97 |
| Infectious dis | 327 | 282 | 294 | 237 | 241 | 214 | 276 | 278 | 199 | 324 | 216 | 2561 | 7.83 |
| Trauma-surgery | 717 | 363 | 449 | 224 | 222 | 262 | 430 | 434 | 161 | 634 | 156 | 3335 | 4.65 |
| Others | 120 | 76 | 89 | 66 | 66 | 59 | 89 | 89 | 49 | 113 | 73 | 769 | 6.41 |
| Pneumonia | 150 | 111 | 126 | 97 | 94 | 73 | 120 | 117 | 99 | 150 | 112 | 1099 | 7.33 |
| Cardiovascular | 188 | 153 | 173 | 121 | 119 | 103 | 161 | 161 | 112 | 188 | 132 | 1423 | 7.57 |
| Total | 2580 | 1726 | 1962 | 1268 | 1266 | 1252 | 1879 | 1885 | 1027 | 2418 | 1276 | 15959 | 6.18 |

Supplemental Table 2c. Frequency of blood examinations performed during the first 24 h, by admission

| Admission | patients | BG | Crea | AST | ALT | Ca | Na | K | CRP | Hgb | Albumin | Total | Per patient |
| --- | --- | --- | --- | --- | --- | --- | --- | --- | --- | --- | --- | --- | --- |
| Elective | 806 | 462 | 514 | 343 | 342 | 340 | 506 | 508 | 196 | 662 | 236 | 4109 | 5.09 |
| Emergency | 1774 | 1264 | 1448 | 925 | 924 | 912 | 1373 | 1377 | 831 | 1756 | 1040 | 11850 | 6.98 |
| Total | 2580 | 1726 | 1962 | 1268 | 1266 | 1252 | 1879 | 1885 | 1027 | 2418 | 1276 | 15959 | 6.18 |

BG = blood glucose; crea = creatinine; AST = aspartate transaminase; ALT = alanine transaminase; Ca = calcium; Na = sodium; K = potassium; CRP = c-reactive protein; Hgb = Hemoglobin

Hemoglobin is used as a proxy of full blood count

**Supplemental Table 3.** Differences between type of admission

|  | **Elective** | **Emergency** | ***p**** |
| --- | --- | --- | --- |
| Total number of patients | 806 | 1,774 |  |
| Male sex | 447 | 870 | 0.0025 |
| Above age threshold | 338 | 1,176 | <.0001 |
| Long duration of in-stay | 201 | 811 | <.0001 |
| Total diseases |  |  | <.0001 |
| 1 | 464 | 517 |  |
| 2 | 207 | 567 |  |
| 3 | 98 | 551 |  |
| >3 | 37 | 139 |  |
| Charlson quartiles |  |  | <.0001 |
| 1 | 293 | 378 |  |
| 2 | 261 | 710 |  |
| 3 | 59 | 378 |  |
| 4 | 193 | 308 |  |
| Classes of disease (primary diagnosis) |  |  | <.0001 |
| Internal medicine | 78 | 478 |  |
| Neoplasia | 311 | 211 |  |
| Infectious diseases | 66 | 261 |  |
| Trauma-surgery | 322 | 395 |  |
| Others | 19 | 101 |  |
| Pneumonia | 4 | 146 |  |
| Cardiovascular | 6 | 182 |  |
| Diabetes (BG>160 mg/dl) § | 38 | 148 | 0.0010 |
| Diabetes (BG>200 mg/dl) §§ | 17 | 103 | <.0001 |
| Low lymphocytes | 410 | 1,115 | 0.4694 |
| High creatinine | 77 | 446 | <.0001 |
| Low albumin | 96 | 570 | <.0001 |
| Low blood glucose | 286 | 845 | 0.0556 |
| Low PNI | 96 | 571 | <.0001 |
| **Dead** | **135** | **785** | <.0001 |

Thresholds for continuous variables are their mean values: age = 66.8 years; duration = 8.8 days; creatinine = 1.15 mg/dl; lymphocytes = 1.6x1000xmm^3^; albumin = 3.2 g/dl; blood glucose = 106.7mg/dl; PNI = 32.4. BG = blood glucose; CRP = c-reactive protein; PNI = prognostic nutrition index.

§ diagnosis of diabetes based on blood glucose levels >160 mg/dl (plus subjects identified in the charts);

§§ diagnosis of diabetes based on blood glucose levels >200 mg/dl (plus subjects identified in the charts);

*Chi Square test

**Supplemental Table 4**. Details of univariate analysis of risk factors for 3-years mortality in the whole sample and in different classes of disease.

|  |  | ***Classes of Disease (Primary Diagnosis)*** | | | | | | |
| --- | --- | --- | --- | --- | --- | --- | --- | --- |
|  | All patients | Internal medicine | Neoplasia | Infectious diseases | Trauma-surgery | Others | Pneumonia | Cardiovascular |
| Number of patients | 2580 | 556 | 522 | 327 | 717 | 120 | 150 | 188 |
| ***Risk Factors*** |  | | | | | | | |
| Type of Admission (urgency vs elective) | **3.26(2.72-3.92); <.0001** | **3.21(1.79-5.74); <.0001** | **4.81(3.65-6.33); <.0001** | **2.66(1.46-4.83); 0.0013** | **3.56(2.18-5.81); <.0001** | 1.65(0.65-4.18); 0.2904 | 0.77(0.24-2.42); 0.6506 | 0.94(0.35-2.56); 0.9089 |
| Total Diseases | **1.46(1.38-1.54); <.0001** | **1.36(1.20-1.55); <.0001** | **1.45(1.31-1.60); <.0001** | **1.31(1.11-1.53); 0.001** | **1.60(1.38-1.86); <.0001** | 1.35(0.97-1.88); 0.0792 | 1.22(0.98-1.50); 0.0724 | **1.28(1.04-1.57); 0.0212** |
| Age-adjusted Charlson index | **1.34(1.31-1.38); <.0001** | **1.56(1.42-1.72); <.0001** | **1.35(1.29-1.41); <.0001** | **1.16(1.09-1.24); <.0001** | **1.84(1.65-2.06); <.0001** | **1.91(1.56-2.33); <.0001** | **1.28(1.15-1.43); <.0001** | **1.35(1.10-1.66); 0.0042** |
| Number of admissions | **1.45(1.19-1.76); 0.0002** | 0.98(0.61-1.56); 0.9288 | 1.20(0.78-1.85); 0.3974 | **1.76(1.13-2.73); 0.0127** | **2.36(1.22-4.55); 0.0105** | 0.82(0.29-2.31); 0.7011 | **2.45(1.33-4.54); 0.0043** | 1.00(0.54-1.84); 0.9861 |
| Sex (F vs M) | 0.94(0.83-1.07); 0.3388 | 1.04(0.79-1.36); 0.7778 | **0.62(0.48-0.81); 0.0005** | 1.35(0.94-1.93); 0.1067 | 0.95(0.64-1.41); 0.7953 | 1.28(0.72-2.30); 0.4019 | 1.20(0.80-1.81); 0.3732 | 0.93(0.65-1.32); 0.6812 |
| Age | **1.06(1.05-1.06); <.0001** | **1.06(1.05-1.07); <.0001** | **1.05(1.03-1.06); <.0001** | **1.05(1.04-1.07); <.0001** | **1.08(1.06-1.10); <.0001** | **1.08(1.05-1.12); <.0001** | **1.04(1.02-1.06); <.0001** | **1.04(1.02-1.06); 0.0001** |
| *Age threshold* | **5.40(4.50-6.49); <.0001** | **4.59(3.10-6.80); <.0001** | **2.74(1.99-3.77); <.0001** | **5.34(3.56-8.00); <.0001** | **12.83(6.67-24.66); <.0001** | **16.05(3.89-66.29); 0.0001** | **2.86(1.48-5.52); 0.0017** | **6.75(1.67-27.29); 0.0074** |
| Duration of in-stay | **1.03(1.02-1.04); <.0001** | 1.02(1.00-1.05); 0.0722 | **1.04(1.02-1.05); <.0001** | 1.01(0.98-1.04); 0.4701 | **1.05(1.04-1.06); <.0001** | 0.98(0.92-1.05); 0.6128 | 1.00(0.97-1.03); 0.9186 | 1.03(0.98-1.09); 0.2375 |
| *Threshold duration* | **1.61(1.42-1.83); <.0001** | 1.19(0.90-1.57); 0.232 | **1.83(1.41-2.37); <.0001** | 1.25(0.87-1.79); 0.2304 | **3.65(2.42-5.49); <.0001** | 0.93(0.39-2.19); 0.8647 | 0.92(0.60-1.40); 0.6921 | 1.20(0.84-1.71); 0.3201 |
| Creatinine* | **1.01(1.01-1.02); <.0001** | **1.01(1.01-1.02); 0.0006** | **1.02(1.00-1.03); 0.0472** | **1.01(1.00-1.03); 0.0445** | 1.01(0.99-1.02); 0.5114 | 1.00(0.97-1.03); 0.9590 | 1.02(0.99-1.04); 0.1133 | **1.02(1.00-1.04); 0.0119** |
| *Threshold creatinine* | **1.92(1.66-2.22); <.0001** | **1.77(1.31-2.39); 0.0002** | **1.81(1.32-2.48); 0.0002** | **1.88(1.23-2.86); 0.0034** | 1.64(0.99-2.72); 0.0569 | 1.60(0.80-3.22); 0.1868 | **1.70(1.10-2.63); 0.0178** | **1.60(1.10-2.31); 0.0133** |
| ALP** | **1.02(1.01-1.03); <.0001** | 1.01(0.99-1.03); 0.2501 | **1.02(1.01-1.03); 0.0007** | **1.03(1.00-1.06); 0.0368** | 1.02(0.99-1.05); 0.1074 | **1.06(1.02-1.10); 0.0020** | 1.03(0.99-1.07); 0.1718 | 1.03(0.95-1.12); 0.4362 |
| gGT** | **1.01(1.01-1.02); 0.0001** | 1.01(0.99-1.02); 0.2156 | **1.01(1.00-1.02); 0.0027** | **1.02(1.01-1.03); 0.0004** | 1.01(0.96-1.06); 0.7784 | 1.01(0.93-1.09); 0.9021 | 1.01(0.98-1.04); 0.6624 | 0.96(0.90-1.02); 0.1596 |
| CHE** | **0.998(0.997-0.999); <.0001** | 0.999(0.997-1.00); 0.0832 | **0.999(0.997-0.999); 0.0397** | **0.995(0.993-0.998); 0.0003** | **0.996(0.993-0.999); 0.0053** | 0.999(0.995-1.00); 0.6079 | 0.998(0.994-1.00); 0.2816 | 0.998(0.996-1.00); 0.0626 |
| Calcium | **0.64(0.56-0.74); <.0001** | **0.57(0.42-0.77); 0.0002** | **0.71(0.55-0.91); 0.0068** | **0.62(0.44-0.86); 0.0039** | 0.71(0.45-1.12); 0.1401 | **0.42(0.21-0.87); 0.0197** | 1.17(0.77-1.76); 0.4641 | **0.42(0.26-0.68); 0.0005** |
| HGB | **0.86(0.84-0.89); <.0001** | **0.87(0.82-0.92); <.0001** | **0.93(0.87-0.98); 0.0092** | **0.81(0.75-0.88); <.0001** | **0.90(0.82-0.98); 0.0145** | 0.93(0.82-1.07); 0.3043 | **0.89(0.80-0.99); 0.0277** | **0.87(0.81-0.94); 0.0004** |
| RBC | **0.65(0.59-0.70); <.0001** | **0.60(0.50-0.70); <.0001** | 0.92(0.77-1.10); 0.3684 | **0.56(0.45-0.70); <.0001** | **0.59(0.46-0.75); <.0001** | 1.06(0.71-1.57); 0.7821 | **0.73(0.53-0.98); 0.0385** | **0.71(0.56-0.89); 0.0026** |
| HCT | **0.96(0.95-0.97); <.0001** | **0.96(0.94-0.98); <.0001** | **0.97(0.95-0.99); 0.0133** | **0.94(0.91-0.96); <.0001** | 0.97(0.94-1.00); 0.0631 | 1.00(0.95-1.05); 0.9415 | 0.97(0.94-1.01); 0.167 | **0.95(0.93-0.98); 0.0003** |
| MCV | **1.03(1.02-1.04); <.0001** | **1.04(1.02-1.06); <.0001** | 1.01(0.99-1.02); 0.4353 | **1.03(1.01-1.05); 0.0086** | **1.08(1.05-1.12); <.0001** | 0.99(0.96-1.02); 0.4648 | 1.03(0.99-1.06); 0.085 | 1.00(0.98-1.02); 0.725 |
| WBC | **1.03(1.02-1.04); <.0001** | 0.99(0.96-1.03); 0.6944 | **1.03(1.01-1.04); <.0001** | **1.02(1.00-1.05); 0.0487** | 1.02(0.97-1.07); 0.4815 | **1.11(1.02-1.20); 0.0183** | **1.04(1.02-1.05); 0.0002** | 1.00(0.96-1.04); 0.9774 |
| Lymphocytes | 1.01(0.98-1.05); 0.4651 | 0.88(0.76-1.03); 0.1222 | 1.02(0.99-1.06); 0.1525 | 0.89(0.76-1.05); 0.1817 | **0.64(0.47-0.87); 0.0046** | 0.65(0.42-1.00); 0.0522 | 1.08(0.90-1.30); 0.4027 | **1.03(1.00-1.06); 0.0364** |
| *Threshold* *Lymphocytes* | **0.66(0.57-0.76); <.0001** | **0.60(0.45-0.81); 0.0007** | 0.97(0.73-1.28); 0.8149 | **0.60(0.40-0.91); 0.0172** | **0.55(0.35-0.86); 0.0094** | **0.47(0.25-0.89); 0.0200** | 1.10(0.69-1.77); 0.6851 | **0.60(0.41-0.90); 0.0133** |
| Albumin | **0.52(0.46-0.59); <.0001** | **0.44(0.33-0.58); <.0001** | **0.76(0.61-0.95); 0.0153** | **0.39(0.28-0.56); <.0001** | **0.51(0.35-0.75); 0.0005** | **0.40(0.21-0.74); 0.0036** | **0.53(0.33-0.84); 0.0073** | **0.49(0.30-0.80); 0.0043** |
| *Threshold Albumin* | **0.49(0.42-0.58); <.0001** | **0.43(0.3-0.61); <.0001** | **0.69(0.50-0.95); 0.0221** | **0.32(0.19-0.55); <.0001** | 0.58(0.31-1.08); 0.0834 | 0.52(0.26-1.07); 0.0757 | 0.55(0.30-1.01); 0.0551 | **0.57(0.37-0.89); 0.0124** |
| Blood Glucose | **1.02(1.00-1.03); 0.0483** | 1.01(0.99-1.04); 0.4315 | 1.00(0.96-1.04); 0.9021 | 1.04(0.99-1.08); 0.1129 | 1.04(0.97-1.11); 0.267 | **1.11(1.00-1.24); 0.0468** | 1.04(0.99-1.10); 0.1428 | 1.02(0.97-1.08); 0.3708 |
| *Threshold* BG | 1.13(0.97-1.32); 0.1131 | **1.40(1.01-1.94); 0.0426** | 0.77(0.57-1.04); 0.088 | 1.29(0.83-1.98); 0.2541 | 1.59(0.99-2.57); 0.0569 | 1.27(0.61-2.66); 0.5255 | 1.21(0.75-1.97); 0.4291 | 1.10(0.72-1.68); 0.6718 |
| PNI | **0.94(0.93-0.95); <.0001** | **0.92(0.89-0.95); <.0001** | **0.97(0.95-0.99); 0.0155** | **0.91(0.88-0.94); <.0001** | **0.94(0.90-0.97); 0.0005** | **0.91(0.86-0.97); 0.0036** | **0.94(0.90-0.98); 0.0081** | **0.93(0.89-0.98); 0.0043** |
| *Threshold PNI* | **0.49(0.42-0.58); <.0001** | **0.43(0.30-0.61); <.0001** | **0.69(0.50-0.95); 0.0221** | **0.33(0.19-0.56); <.0001** | 0.59(0.32-1.09); 0.0893 | 0.52(0.26-1.07); 0.0757 | **0.51(0.27-0.96); 0.0364** | **0.57(0.37-0.89); 0.0124** |
| Diabetes (BG>160 mg/dl) § | **1.53(1.23-1.91); 0.0002** | 1.20(0.80-1.82); 0.3767 | 1.43(0.89-2.29); 0.1352 | 1.59(0.91-2.79); 0.1016 | **2.14(1.04-4.42); 0.0385** | 1.88(0.45-7.76); 0.3840 | 1.64(0.79-3.38); 0.1846 | 1.24(0.63-2.44); 0.5403 |
| Diabetes (BG>200 mg/dl) §§ | **1.49(1.14-1.95); 0.0038** | 1.10(0.70-1.72); 0.6853 | **3.14(1.71-5.77); 0.0002** | 1.32(0.62-2.84); 0.4745 | 1.86(0.68-5.04); 0.2262 | N.E. | 1.20(0.56-2.59); 0.6419 | 0.83(0.36-1.87); 0.6457 |

Data are presented as Hazard Ratios (95% confidence intervals); p-values. Hazard Ratio (HR) for continuous variables were calculated for 1-unit increase. Significant values are in bold. Thresholds for variables are their mean values: age = 66.8 years; duration = 8.8 days; creatinine = 1.15 mg/dl; lymphocytes = 1.6x1000xmm^3^; albumin = 3.2 g/dl; blood glucose = 106.7mg/dl; PNI = 32.4.

ALP = alkaline phosphatase; gGT = gamma-glutamil-transpeptidase; CHE = cholinesterase; HGB = hemoglobin; RBC = red blood cells; HCT = hematocrit; MCV = mean corpuscular volume; WBC = white blood count; BG = blood glucose; PNI = prognostic nutritional index.

*HR for 10-unit increase; ** HR for 0.1-unit increase; § diagnosis of diabetes based on blood glucose levels >160 mg/dl (plus subjects identified in the charts); §§ diagnosis of diabetes based on blood glucose levels >200 mg/dl (plus subjects identified in the charts);

**Supplemental Table 5.** Age-adjusted Charlson Index in alive and dead patients

|  | **Age-adjusted charlson Index** | |  |
| --- | --- | --- | --- |
| **Classes of disease**  **(primary diagnosis)** | **Patients alive** | **Patients dead** | ***p**** |
| Internal medicine | 3.2±1.75 | 4.6±1.10 | <0.0001 |
| Neoplasia | 4.4±2.42 | 7.4±2.73 | <0.0001 |
| Infectious diseases | 3.2±2.78 | 4.7±2.02 | <0.0001 |
| Trauma-surgery | 2.2±1.66 | 4.1±1.08 | <0.0001 |
| Others | 2.5±1.91 | 4.8±1.22 | <0.0001 |
| Pneumonia | 3.3±1.59 | 4.5±1.34 | <0.0001 |
| Cardiovascular | 4.8±0.85 | 5.3±0.79 | 0.0002 |
| **Total** | **3.1±2.16** | **5.4±2.15** | **<0.0001** |

Means ± standard deviation. *Wilcoxon test

**Supplemental Table 6.** Details of multivariable Cox regression models to evaluate variables predicting mortality.

|  | **Classes of disease (primary diagnosis)** | | | | | | | |
| --- | --- | --- | --- | --- | --- | --- | --- | --- |
|  | All patients | Internal medicine | Neoplasia | Infectious diseases | Trauma-surgery | Others | Pneumonia | Cardiovascular |
| **Model 1.** |  | | | | | | | |
| Age-adjusted Charlson index | **1.25(1.21-1.3); <.0001** | **1.34(1.19-1.52); <.0001** | **1.21(1.14-1.28); <.0001** | **1.14(1.03-1.26); 0.0087** | **1.72(1.29-2.28); 0.0002** | **1.77(1.37-2.29); <.0001** | **1.22(1.04-1.44); 0.0165** | **1.37(1.05-1.79); 0.0192** |
| Type of Admission (urgency) | **2.41(1.88-3.08); <.0001** | 1.59(0.69-3.65); 0.2725 | **2.90(2.05-4.10); <.0001** | **4.83(1.98-11.78); 0.0005** | **3.87(1.38-10.86); 0.01** | 0.86(0.23-3.28); 0.8277 | 0.76(0.18-3.28); 0.7171 | 0.55(0.20-1.51); 0.2455 |
| Number of Admissions | **1.41(1.11-1.78); 0.0051** | 1.39(0.83-2.32); 0.2125 | 1.00(0.54-1.85); 0.9935 | 1.65(0.99-2.73); 0.0514 | **2.28(1.05-4.96); 0.0372** | 1.35(0.34-5.38); 0.6729 | **2.59(1.21-5.51); 0.0138** | 1.14(0.60-2.17); 0.6987 |
| PNI | **0.95(0.94-0.97); <.0001** | **0.93(0.90-0.96); <.0001** | 0.98(0.95-1.00); 0.0799 | **0.94(0.90-0.98); 0.0026** | 0.96(0.92-1.00); 0.0671 | 0.96(0.89-1.04); 0.3048 | 0.96(0.91-1.01); 0.1322 | **0.95(0.90-0.99); 0.0477** |
| Hemoglobin | **0.94(0.91-0.98); 0.0019** | 0.98(0.91-1.05); 0.5787 | 0.99(0.92-1.06); 0.7691 | **0.83(0.74-0.92); 0.0005** | 0.99(0.86-1.13); 0.863 | 1.04(0.86-1.24); 0.7057 | 0.91(0.78-1.05); 0.1986 | 0.92(0.83-1.02); 0.1121 |
| **Model 2.** |  | | | | | | | |
| Age | **1.04(1.03-1.04); <.0001** | **1.05(1.03-1.06); <.0001** | 1.01(0.99-1.03); 0.077 | **1.04(1.03-1.06); <.0001** | **1.07(1.04-1.11); <.0001** | **1.08(1.04-1.12); 0.0003** | **1.02(1-1.05); 0.0279** | **1.03(1-1.06); 0.0314** |
| Type of Admission (urgency) | **1.41(1.11-1.81); 0.006** | 1.42(0.62-3.25); 0.4073 | **2.88(2.04-4.07); <.0001** | 2.05(0.85-4.95); 0.1117 | 1.63(0.60-4.46); 0.3398 | 0.65(0.17-2.57); 0.5409 | 0.83(0.20-3.52); 0.8009 | 0.65(0.24-1.78); 0.3963 |
| Number of Admissions | **1.49(1.17-1.89); 0.0011** | 1.32(0.78-2.22); 0.3066 | 0.94(0.51-1.74); 0.8552 | **2.24(1.28-3.91); 0.0045** | **4.58(2.07-10.15); 0.0002** | 1.39(0.29-6.74); 0.6866 | **2.57(1.2-5.5); 0.0152** | 1.11(0.59-2.12); 0.7434 |
| PNI | **0.96(0.94-0.97); <.0001** | **0.93(0.90-0.96); <.0001** | 0.98(0.96-1.01); 0.2067 | **0.95(0.91-0.99); 0.0397** | 0.97(0.92-1.01); 0.1042 | 0.96(0.88-1.03); 0.2404 | 0.96(0.91-1.01); 0.1151 | 0.96(0.91-1.02); 0.1849 |
| Hemoglobin | **0.94(0.91-0.98); 0.0022** | 0.98(0.91-1.06); 0.5677 | 0.97(0.90-1.04); 0.3858 | **0.87(0.78-0.97); 0.0097** | 0.98(0.85-1.12); 0.748 | 1.04(0.87-1.24); 0.6888 | 0.9(0.78-1.03); 0.1289 | 0.91(0.82-1.01); 0.0621 |

Data are presented as Hazard Ratios for 1-unit increase (95% confidence intervals); p-values. Significant values are in bold. Showed variables were selected as best model from two stepwise regression models in the whole population. To avoid collinearity, model 1 included age-adjusted Charlson index, while in model 2 age plus total diseases were used (total diseases was not selected from the stepwise procedure). Selected variables in the whole population were tested as multivariable models for each class of disease.

PNI=prognostic nutritional index
